# Supplementary material for: Longitudinal Analysis of Placental IRS1 DNA Methylation and Childhood Obesity
Source: Int J Mol Sci. 2025 Mar 28;26(7):3141. doi: 10.3390/ijms26073141 (PMC11988732; doi:10.3390/ijms26073141)
Supplement: Supplementary file 1 [file ijms-26-03141-s001.zip › Supplementary methods.pdf]

Supplementary File.

Supplementary Methods

Inclusion and exclusion criteria

All women included in the study were of Caucasian origin, delivered infants born at term (37 to 40 weeks) from singleton pregnancies. The exclusion criteria were: women with medical, surgical, or obstetrical complications including pregestational and/or gestational metabolic diseases (pregestational and/or gestational diabetes, hypertension and/or preeclampsia, glucose intolerance) or fetal growth restriction; newborn malformations or asphyxia; assisted reproductive technology; and alcohol abuse or drug addiction during pregnancy.

Biological samples collection and handling

Immediately after childbirth, four placenta tissue biopsies (one from each quadrant) were obtained from the maternal side of the placenta after the decidua was removed. Samples were washed with saline buffer to remove blood and were stored at −80ºC. Peripheral blood samples from the children at 6 years of age were collected in fasting conditions. The leucocyte fraction was obtained after blood centrifugation and was stored at -80ºC.

Infant's anthropometric assessments and derived variable calculations

Weight was measured on a calibrated scale wearing light clothes, and height was measured with a Harpenden stadiometer without shoes. BMI and age- and sex-adjusted z-scores were calculated as above mentioned. Waist circumference was measured in the supine position between the last rib and the iliac crest, while hip circumference was measured in the standing position at the level of the greater trochanters. The change between weight at birth and at 6 years (Δ BW-SDS to weight-SDS) was calculated as the subtraction between weight-SDS at 6 years and birth weight (BW-SDS). Fat mass (FM) was assessed by bioelectric impedance (Hydra Bioimpedance Analyzer 4200; Xitron Technologies, San Diego CA, USA) and it was calculated as body weight minus

lean body mass (LBM). An electronic oscillometer (Dinamap®Pro 100; GE Medical Systems, Chalfont St. Giles, UK) with appropriate cuff size was used to measure blood pressure. It was measured on the right arm, after a 10-minute rest, and the patient was in the supine position. Data are presented as the average of two measurements.

Fat distribution was measured by high-resolution ultrasound (MyLabTM25, Esaote, Firenze, Italy). The children were placed in the supine position and the probe was kept perpendicular to the skin. Subcutaneous and preperitoneal fat were assessed at the subxiphoid level using a linear 7.5–12 MHz transducer and visceral fat was assessed at the abdominal level using a convex 3–3.5 MHz transducer as described by Hirooka et. al. [1]. Averages of three measurements were used in the study. All measurements were performed by the same operator who was blinded to treatment allocation. The intra-subject coefficient variation was less than 6%.

Serum glucose was measured by the hexokinase method. Insulin was measured by immunochemiluminiscence (IMMULITE 2000, Diagnostic Products, Los Angeles, CA, USA). The lower detection limit was 0.4 mIU/L, and the intra- and inter-assay coefficients of variation (CVs) were <10%. Insulin resistance was estimated from fasting insulin and glucose levels using the homeostasis model assessment [HOMA-IR = (fasting insulin in mIU/L) × (fasting glucose in mM)/22.5]. Total serum triacylglycerol (TG) was measured by monitoring the reaction of glycerol-phosphate oxidase (ARCHITECT, Abbott Laboratories, Abbott Park, IL, USA); the lower detection limit was 5.0 mg/dL and intra- and inter-assay CVs <5%. High-density lipoprotein cholesterol (HDL-cholesterol) was quantified by a homogenous method of selective detergent with accelerator (ARCHITECT, Abbott Laboratories, Abbott Park, IL, USA); the lower detection limit was 2.5 mg/dL and intra- and inter-assay CVs <4%.

Data normalization and statistical analysis of the Infinium MethylationEPIC BeadChip microarray data

Raw IDAT files were normalized using functional normalization with R package minfi (version 1.28.0). β-values (in representation of the methylation level) and a detection p-value (in representation of the confidence of a given β-value) were provided for each CpGs. The CpGs on the microarray with a detection p-value > 0.01, those targeting SNPs and those located in sexual chromosomes were removed.

The differentially methylated CpGs (DMCs) associated with offspring BMI were identified using Beta Regression Models, using offspring BMI-SDS as a predictor and the methylation level of each CpGs as a response. Hypermethylation was referred when a positive relationship was observed between methylation and offspring BMI-SDS and hypomethylation was referred when a negative relationship was observed between methylation and offspring BMI-SDS. To correct for multiple comparisons, p-values were subsequently adjusted using False Discovery Rate (FDR). All statistical analyses were performed using R (version 3.5.1) and R package betareg (version 2.0-13). Adjusted p-values < 0.05 were considered statistically significant. Given that offspring BMI-SDS is a continuous variable ranging from -1.39 to 3.13 in the screening sample, Odds Ratio (OR) was used as a measure of biological effect. CpGs with OR values greater than 1.25 or lower than 0.8 were considered more relevant. Methylation raw data from the microarray have been deposited in the Gene Expression Omnibus database (accession number GSE192812).

DNA methylation assessment

Genomic DNA was isolated from both placenta samples and leucocytes from blood samples using the QIAmp DNA Mini Kit (QIAGEN, Germany). Then, DNA bisulfite conversion was performed using the EpiTect Fast DNA Bisulfite Kit (QIAGEN, Germany). Bisulfite converted DNA (bs-DNA) was amplified by real-time PCR using specific amplifying primers (**Table S1**). Pyrosequencing was performed using PyroMark Q48 instrument (QIAGEN, Germany) and working with specific pyrosequencing primers (**Table S1**). Raw data were analysed using the Pyromark Q48 Autoprep Software V2.4.2

(QIAGEN, Germany) to obtain the percentage of methylation for each CpGs. Further details on procedure are described elsewhere [2].

Gene expression assessment

Total RNA from both leucocyte and placenta samples was isolated using the RNeasy Mini Kit (QIAGEN, Germany), and subsequently reverse-transcribed with the High-Capacity cDNA Reverse Transcription Kit (Applied Biosystems). Then, cDNA was amplified using the commercially available TaqMan Gene Expression assay *IRS1* (Hs00178563_m1). Reactions were run on a LightCycler 480 Real-Time PCR System (Roche Diagnostics, Rotkreuz, Switzerland), using the default cycling conditions. Relative gene expression levels were calculated according to the $2^{-\Delta Ct}$ method, using the average values obtained by endogenous controls for placenta samples [TaqMan Gene Expression assays *SDHA* (Hs00188166_m1) and *TBP* (Hs00427620_m1)].

Statistical analysis

In the validation analysis, anthropometric and metabolic variables are presented as mean ± standard error of the mean (SEM). Continuous variables were tested for normality; those non-normally distributed were either log-transformed or analyzed using non-parametric tests. Unpaired t-tests were used to assess differences between BMI-SDS 50th percentile groups. The *IRS1* methylation and expression levels were both studied in placenta and in blood samples from the offspring at 6 years of age, and all were correlated with offspring's metabolic parameters at 6 years (**Figure S1**). Associations between variables were assessed using Pearson correlation analysis, followed by multiple regression analyses to adjust for potential confounding factors (offspring age and sex). The significance level was set at $p \leq 0.05$.

Prediction analysis

Predictive models were developed using ML analyses, incorporating clinical and anthropometric parameters from the study cohort during prenatal (pre-pregnancy and pregnancy) and neonatal (birth and 12 months of age) periods. Initially, non-null instances for the IRS1 variable were selected to ensure data integrity. The target variable BMI-SDS was used to classify the samples into two groups (BMI-SDS<p50 and BMI-SDS>p50). Variables with more than 35% missing values were excluded to enhance the quality of the analysis. The remaining variables were imputed using the KNN (k-nearest neighbors) algorithm and adjusted for correlation to prevent multicollinearity. Subsequently, the data set was divided into training and testing sets (70% and 30%, respectively). The most influential features were identified using the f_classif algorithm, and these variables together with methylation and expression data were included in the analysis. We used the Extra Trees model and was evaluated with performance metrics including recall, precision, accuracy and F1-score. Finally, the Shapley package was employed to interpret the results, generating a plot to visualize the impact of the variables on the model's predictions and their individual trends.

References

1. Hirooka M, Kumagi T, Kurose K, Nakanishi S, Michitaka K, Matsuura B, et al. A technique for the measurement of visceral fat by ultrasonography: comparison of measurements by ultrasonography and computed tomography. Intern Med. 2005 Aug;44(8):794-9. doi: 10.2169/internalmedicine.44.794. PMID: 16157975

2. Gómez-Vilarrubla A, Mas-Parés B, Carreras-Badosa G, Xargay-Torrent S, Prats-Puig A, Bonmatí-Santané A, de Zegher F, Ibañez L, López-Bermejo A, Bassols J. Placental epigenetic marks related to gestational weight gain reveal potential genes associated with offspring obesity parameters. Obesity (Silver Spring). 2023 Jul;31(7):1903-1912. doi: 10.1002/oby.23780. Epub 2023 Jun 14. PMID: 37313637.
