# Supplementary material for: Longitudinal Analysis of Placental IRS1 DNA Methylation and Childhood Obesity
Source: Int J Mol Sci. 2025 Mar 28;26(7):3141. doi: 10.3390/ijms26073141 (PMC11988732; doi:10.3390/ijms26073141)
Supplement: Supplementary file 1 [file ijms-26-03141-s001.zip › Table S1.pdf]

Table S1. Pyrosequencing primers design.

Gene		Primers	Temp (ºc)	Size (bp)
IRS1_1	Fw	**BIOT**- TGTATGGAGTTTAGGGTATTTTTAAGTTA	56	262
	Rv	CCCATTTTATTTCCCTTCTCTCT		
	Seq	CCCTTCTCTCTTTACT		
IRS1_2	Fw	GTAGTAAAGAGAGAAGGGAAATAAAATGG	56	255
	Rv	**BIOT**- ACCCTACCTAACACCTCTTTC		
	Seq	TTAAATGTAAGAATATAGTTGAG		
IRS1_3	Fw	**BIOT**- GGTGTTAGGTAGGGTGGTAGAA	56	131
	Rv	CCCACCACAACCAATCAACATA		
	Seq	CCACAACCAATCAACATAT		

IRS1_1 corresponds to the primer design to amplify a gene region including the first CpGs (IRS1 CpG1); IRS1_2 corresponds to the primer design to amplify a gene region including the second and third CpGs (IRS1 CpG2 and IRS1 CpG3); IRS1_3 corresponds to the primer design to amplify a gene region including the fourth CpGs (IRS1 CpG4); Fw: Forward primer; Rv: Reverse primer; Seq: Sequence primer; Temp: Annealing temperature; Size: Amplified fragment size; bp: Base pairs.
