# Supplementary material for: Longitudinal Analysis of Placental IRS1 DNA Methylation and Childhood Obesity
Source: Int J Mol Sci. 2025 Mar 28;26(7):3141. doi: 10.3390/ijms26073141 (PMC11988732; doi:10.3390/ijms26073141)
Supplement: Supplementary file 1 [file ijms-26-03141-s001.zip › Table S2.pdf]

Table S2. Clinical characteristics of the subjects from the screening and validation groups separated by sex.

	Screening (n=24)			Validation (n=147)		
	Girls	Boys	p-value	Girls	Boys	p-value
**Mother (n)**	12	12		77	70	
Age (yr)	32 ± 1	29 ± 1	Ns	31 ± 1	31 ± 1	Ns
Height (cm)	166 ± 2	164 ± 2	Ns	162 ± 1	163 ± 1	Ns
Pregestational weight (kg)	69.8 ± 4.6	67.2 ± 3.7	Ns	64.9 ± 1.5	66.3 ± 1.4	Ns
Pregestational BMI (Kg/m^2)	25.4 ± 1.8	25.0 ± 1.2	Ns	24.4 ± 0.5	24.9 ± 0.5	Ns
Pregestational Obesity (%)	33	33	Ns	42	33	Ns
**Newborn (n)**	12	12		77	70	
Gestational age (wk)	40 ± 0.2	40 ± 0.2	Ns	40 ± 0.1	40 ± 0.1	Ns
Birth weight (Kg)	3.4 ± 0.1	3.4 ± 0.1	Ns	3.3 ± 0.1	3.4 ± 0.1	Ns
Birth weight-SDS	0.2 ± 0.2	0.3 ± 0.2	Ns	0.2 ± 0.1	0.2 ± 0.1	Ns
Birth length(cm)	50.8 ± 0.3	50.2 ± 0.4	Ns	49.4 ± 0.2	50.1 ± 0.1	Ns
Birth length-SDS	0.07 ± 0.2	0.06 ± 0.2	Ns	-0.02 ± 0.1	0.05 ± 0.1	Ns
**Offspring at 6 yr (n)**	12	12		77	70	
Age (yr)	6.4 ± 0.1	6.1 ± 0.1	Ns	6.0 ± 0.1	5.8 ± 0.1	Ns
Weight (kg)	22.5 ± 0.9	24.9 ± 1.6	Ns	22.2 ± 0.5	22.6 ± 0.6	Ns
Weight SDS	-0.15 ± 0.2	0.59 ± 0.4	Ns	0.22 ± 0.2	0.05 ± 0.1	Ns
Height (cm)	119 ± 1	120 ± 2	Ns	116 ± 1	116 ± 1	Ns
Height-SDS	0.16 ± 0.3	1.08 ± 0.4	Ns	-0.01 ± 0.1	0.25 ± 0.1	Ns
BMI (kg/m^2)	15.7 ± 0.4	16.9 ± 0.6	Ns	16.2 ± 0.2	16.5 ± 0.2	Ns
BMI-SDS	-0.28 ± 0.2	0.22 ± 0.3	Ns	-0.02 ± 0.1	0.05 ± 0.1	Ns
Δ BW-SDS to weight-SDS	-0.41 ± 0.3	0.5 ± 0.5	Ns	-0.26 ± 0.1	-0.07 ± 0.1	Ns
Waist (cm)	54.9 ± 1.2	60.0 ± 3.7	Ns	56.0 ± 0.7	56.6 ± 0.9	Ns
Hip (cm)	59.8 ± 1.9	63.0 ± 3.5	Ns	60.1 ± 0.8	59.0 ± 1.0	Ns
SBP (mmHg)	95.9 ± 5.0	98.2 ± 3.1	Ns	95.9 ± 1.3	96.0 ± 1.5	Ns
DBP (mmHg)	58.4 ± 1.3	55.7 ± 1.9	Ns	56.6 ± 0.9	57.6 ± 0.7	Ns
HDL-cholesterol (mg/dl)	54.8 ± 4.2	59.8 ± 3.3	Ns	55.0 ± 1.1	56.8 ± 1.2	Ns
Triglycerides (mg/dl)	52.9 ± 3.2	45.4 ± 4.4	Ns	54.0 ± 1.8	47.4 ± 1.7	Ns
Glucose (mg/dl)	83.7 ± 1.7	86.5 ± 3.1	Ns	82.5 ± 0.8	83.3 ± 0.8	Ns
Insulin (mIU/L)	5.9 ± 0.4	6.5 ± 1.0	Ns	5.5 ± 0.2	4.9 ± 0.2	Ns
HOMA-IR	1.2 ± 0.1	1.4 ± 0.3	Ns	1.1 ± 0.1	1.0 ± 0.1	Ns
FM (Kg)	5.6 ± 0.6	6.4 ± 1.4	Ns	6.1 ± 0.3	5.0 ± 0.3	0.02
LBM (Kg)	16.8 ± 0.6	19.7 ± 1.2	0.02	16.1 ± 0.3	17.9 ± 0.3	<0.001
Subcutaneous fat (cm)	0.44 ± 0.04	0.39 ± 0.06	Ns	0.49 ± 0.03	0.42 ± 0.03	Ns
Peritoneal fat (cm)	0.43 ± 0.06	0.47 ± 0.07	Ns	0.47 ± 0.02	0.44 ± 0.02	Ns
Visceral fat (cm^2)	5.4 ± 0.3	5.5 ± 0.4	Ns	5.4 ± 0.1	5.1 ± 0.1	Ns
**Methylation and expression values**				**77**	**70**	
Placental *IRS1* CpG2 methylation (%)	--	--	--	1.8 ± 0.1	1.7 ± 0.1	Ns
Placental *IRS1* expression ($2^{-\Delta CT}$)	--	--	--	0.62 ± 0.01	0.51 ± 0.06	Ns
Leucocyte *IRS1* CpG2 methylation (%)	--	--	--	1.8 ± 0.1	1.6 ± 0.1	Ns
Leucocyte *IRS1* expression ($2^{-\Delta CT}$)	--	--	--	0.03 ± 0.01	0.02 ± 0.01	Ns

Data are shown as mean ± SEM. BMI: Body mass index; SDS: Standard deviation score; Δ BW-SDS to weigth-SDS: z-score changes from weight at birth to weight at 6 years; SBP: Systolic blood pressure; DBP: Diastolic blood pressure; HDL: High density lipoprotein; HOMA-IR: Homeostatic model assessment for insulin resistance; FM: Fat mass; LBM: Lean body mass; Ns: Non-significant.
