# Supplementary material for: Longitudinal Analysis of Placental IRS1 DNA Methylation and Childhood Obesity
Source: Int J Mol Sci. 2025 Mar 28;26(7):3141. doi: 10.3390/ijms26073141 (PMC11988732; doi:10.3390/ijms26073141)
Supplement: Supplementary file 1 [file ijms-26-03141-s001.zip › Table S4.pdf]

Table S4. Gene set enrichment analysis (KEGG pathways) of DMCs associated with offspring BMI-SDS.

Pathway Name	Overlap	P-value	Adjusted P-value	Genes
mTOR signaling pathway	18/154	5,78E+10	0.01	FZD1;**IRS1**;PRKCB;INSR;WNT7B;LRP5;PIK3R2; PIK3R1;WNT16;IKBKB;RPTOR;SLC7A5;WNT11; RPS6KA2;RPS6KA1;ULK2;LPIN2;TELO2
Type 2 diabetes	09/46	8,41E+10	0.01	IKBKB;**IRS1**;INSR;PDX1;PIK3R2;CACNA1C;PIK3R1; PRKCZ;CACNA1G
Spinocerebellar ataxia	16/143	2,44E+11	0.02	RYR1;XBP1;PRKCB;KCND3;PIK3R2;PIK3R1;RBPJ; PUM2;GRIN1;PSMD8;AFG3L2;ATXN3;PSMB7; DAB1;ULK2;MAP3K5
Phosphatidylinositol signaling system	11/97	0.001	0.14	INPP4A;PI4K2B;INPP4B;INPP5A;PRKCB;PI4KA; PIK3R2; PIK3R1;PIP5K1C;SYNJ2;DGKH
Calcium signaling pathway	19/240	0.004	0.25	RYR1;CHRM2;NTRK1;RYR2;PDGFRA;MCOLN3; PRKCB;PTAFR;ATP2B4;CACNA1C;FGF1;GRIN1; CACNA1G;SLC8A3;EDNRB;ERBB4;PDGFC;DRD1; FGF23
Breast cancer	13/147	0.007	0.30	FZD1;WNT7B;LRP5;PIK3R2;PIK3R1;FGF1;WNT16; ESR1;DDB2;HEYL;WNT11;PGR;FGF23
Progesterone-mediated oocyte maturation	10/100	0.007	0.30	FZR1;HSP90AB1;RPS6KA2;RPS6KA1;ANAPC5;PGR; PIK3R2;PIK3R1;MAD1L1;ANAPC10
MAPK signaling pathway	21/294	0.009	0.30	MAP2K3;NTRK1;PDGFRA;PRKCB;INSR;NFATC1; CACNA1C;FGF1;DUSP6;MAPK8IP1;CACNA1G; RASGRP3; IKBKB;ERBB4;RPS6KA2;RPS6KA1;PDGFC; GNA12;MAP3K8;FGF23;MAP3K5
Inositol phosphate metabolism	08/73	0.009	0.30	INPP4A;PI4K2B;INPP4B;INPP5A;PI4KA;PIP5K1C; SYNJ2; PLCH2
Rap1 signaling pathway	16/210	0.012	0.32	MAP2K3;PDGFRA;RALA;RGS14;PRKCB;INSR; PIK3R2;PIK3R1;FGF1;PRKCZ;GRIN1;RASGRP3;ENA; PDGFC;FGF23;RAPGEF4
Insulin resistance	10/108	0.012	0.32	IKBKB;**IRS1**;PRKCB;RPS6KA2;INSR;RPS6KA1;NR1H3; PIK3R2;PIK3R1;PRKCZ
Ras signaling pathway	17/232	0.014	0.32	NTRK1;PDGFRA;RALBP1;RAB5B;RALA;PRKCB;INSR; PIK3R2;PIK3R1;RASAL2;FGF1;GRIN1;RASGRP3; IKBKB;PDGFC;FGF23;RAB5A

Aldosterone-regulated sodium reabsorption	05/37	0.016	0.32	**IRS1**;PRKCB;INSR;PIK3R2;PIK3R1
Alzheimer disease	24/369	0.016	0.32	FZD1;XBP1;**IRS1**;NDUFA4;INSR;WNT7B;NDUFA4L2;LRP5;PIK3R2;PIK3R1;CACNA1C;WNT16;RTN4;GRIN1;PSMD8;IKBKB;PSMB7;TUBA3E;TUBA3D;TUBB6;WNT11;ULK2;NDUFV1;MAP3K5
Regulation of actin cytoskeleton	16/218	0.017	0.32	CHRM2;PDGFRA;ARHGEF12;PIK3R2;PIK3R1;FGF1;ENAH;FGD3;PDGFC;ITGA11;GNA12;ITGAV;PIP5K1C;FGF23;DOCK1;VCL
Endocrine and other factor-regulated calcium reabsorption	06/53	0.020	0.34	SLC8A3;CALB1;PRKCB;AP2S1;ATP2B4;ESR1
Pathways of neurodegeneration	29/475	0.020	0.34	RYR1;RYR2;LRRK2;NDUFA4L2;LRP5;CACNA1C;PSMD8;PSMB7;TUBA3E;ATXN3;TUBA3D;TUBB6;WNT11;SPG11;NDUFV1;MAP3K5;FZD1;MAP2K3;XBP1;DNAI2;PRKCB;NDUFA4;WNT7B;WNT16;GRIN1;ALS2;ULK2;SQSTM1;RAB5A
Biosynthesis of unsaturated fatty acids	04/27	0.022	0.35	ELOVL1;ACOT7;SCP2;SCD
AMPK signaling pathway	10/120	0.024	0.35	RPTOR;**IRS1**;SCD;INSR;PIK3R2;PIK3R1;EEF2;ADIPOR2;ELAVL1;PFKM
Non-alcoholic fatty liver disease	12/155	0.024	0.35	IKBKB;XBP1;**IRS1**;NDUFA4;INSR;NDUFA4L2;NR1H3;PIK3R2;PIK3R1;ADIPOR2;NDUFV1;MAP3K5
Melanoma	07/72	0.027	0.36	PDGFRA;PDGFC;PIK3R2;PIK3R1;FGF1;FGF23;DDB2
PD-L1 expression and PD-1 checkpoint pathway in cancer	08/89	0.028	0.36	IKBKB;MAP2K3;IFNGR2;PIK3R2;NFATC1;PIK3R1;PDCD1;JAK2
Pathways in cancer	31/531	0.029	0.36	RALA;HSP90AB1;CTBP2;LRP5;PIK3R2;PIK3R1;FGF1;RASGRP3;IKBKB;EDNRB;WNT11;GNA12;ITGAV;CTNNA3;JAK2;FGF23;FZD1;NTRK1;PDGFRA;RALBP1;ARHGEF12;PRKCB;IFNGR2;WNT7B;DAPK3;WNT16;ESR1;DDB2;RUNX1;HEYL;TRAF3
Bacterial invasion of epithelial cells	07/77	0.037	0.42	ARHGAP10;PIK3R2;CTNNA3;PIK3R1;DOCK1;VCL;CD2AP
Riboflavin metabolism	08/24	0.039	0.42	ACP2;ACP1

Cell adhesion molecules	11/148	0.039	0.42	CD86;CDH4;CLDN8;NRXN1;ITGAV;NCAM1;PDCD1;PVR;CD34;GLG1;JAM2
Gastric cancer	11/149	0.041	0.42	FZD1;WNT11;WNT7B;LRP5;PIK3R2;CTNNA3;PIK3R1;FGF1;WNT16;FGF23;DDB2
Base excision repair	04/33	0.043	0.42	PCNA;NEIL2;POLE;UNG
Circadian entrainment	08/97	0.044	0.42	RYR1;PER2;RYR2;MTNR1A;PRKCB;CACNA1C;CACNA1G;GRIN1
GnRH secretion	06/64	0.045	0.42	PRKCB;PIK3R2;KCNN2;CACNA1C;PIK3R1;CACNA1G
Choline metabolism in cancer	08/98	0.046	0.42	PDGFRA;PCYT1A;PRKCB;PDGFC;PIK3R2;PIK3R1;PIP5K1C;DGKH
