# Supplementary material for: Longitudinal Analysis of Placental IRS1 DNA Methylation and Childhood Obesity
Source: Int J Mol Sci. 2025 Mar 28;26(7):3141. doi: 10.3390/ijms26073141 (PMC11988732; doi:10.3390/ijms26073141)
Supplement: Supplementary file 1 [file ijms-26-03141-s001.zip › Table S5.pdf]

Table S5. Logistic Regression Models (LRM) to predict offspring BMI and visceral fat.

**BMI (50th percentile)**	**p-value**	**Beta**	**95% IC**	**R^2**
Placental *IRS1* methylation	0.01	2.32	1.15-4.63	0.076
**Visceral fat (50th percentile)**	**p-value**	**Beta**	**95% IC**	**R^2**
Placental *IRS1* expression	0.03	2.69	1.10-6.60	0.070
Leucocyte *IRS1* expression	0.02	5.23	24.25-112.9	0.096

BMI: body mass index; IC: interval of confidence
