# Supplementary figures and images for: Longitudinal Analysis of Placental IRS1 DNA Methylation and Childhood Obesity
Source: Int J Mol Sci. 2025 Mar 28;26(7):3141. doi: 10.3390/ijms26073141 (PMC11988732; doi:10.3390/ijms26073141)

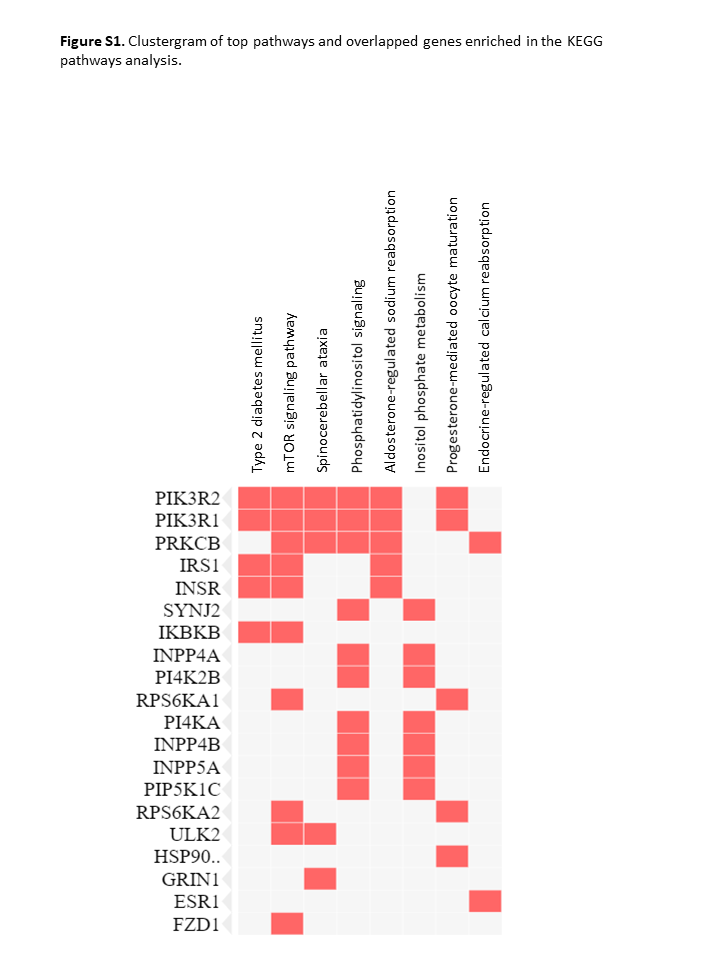

Supplement: Supplementary file 1 [file ijms-26-03141-s001.zip › Figure S1.tif]

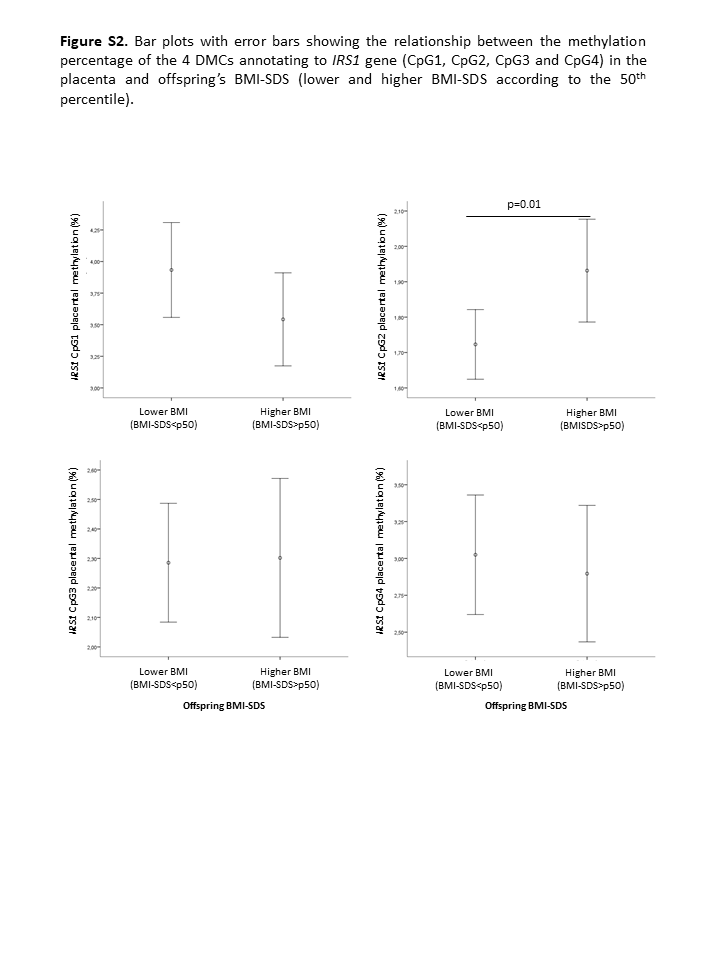

Supplement: Supplementary file 1 [file ijms-26-03141-s001.zip › Figure S2.tif]

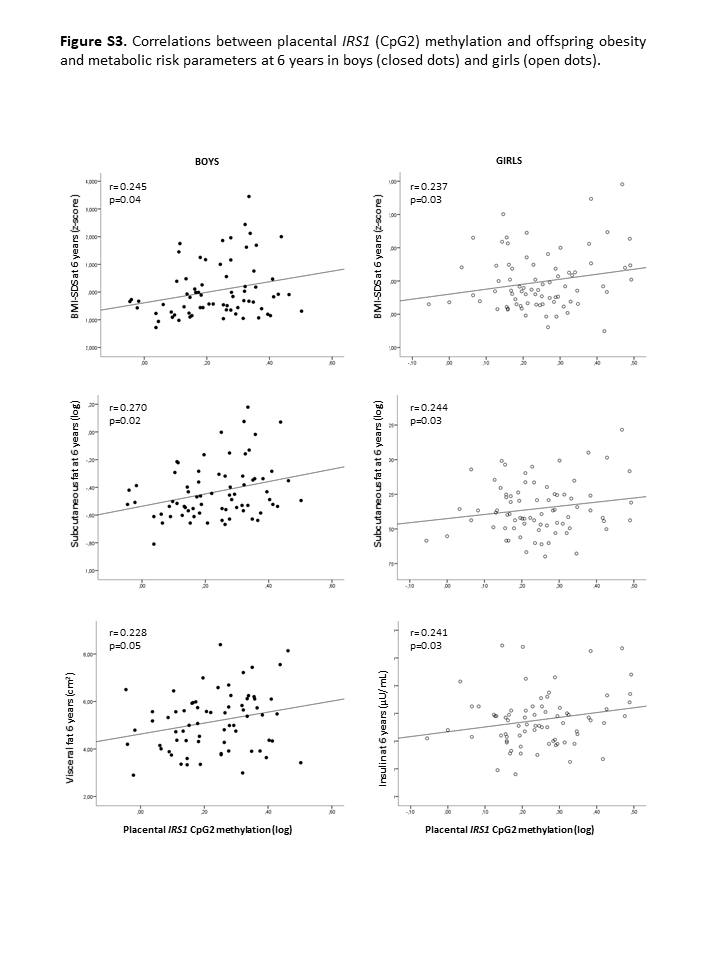

Supplement: Supplementary file 1 [file ijms-26-03141-s001.zip › Figure S3.tif]

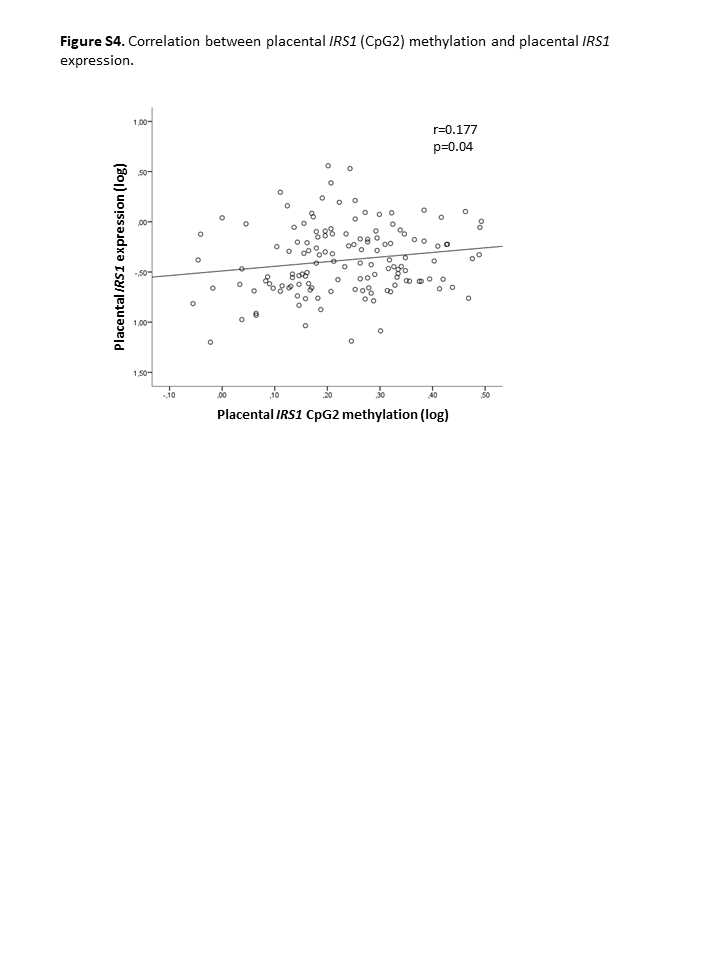

Supplement: Supplementary file 1 [file ijms-26-03141-s001.zip › Figure S4.tif]

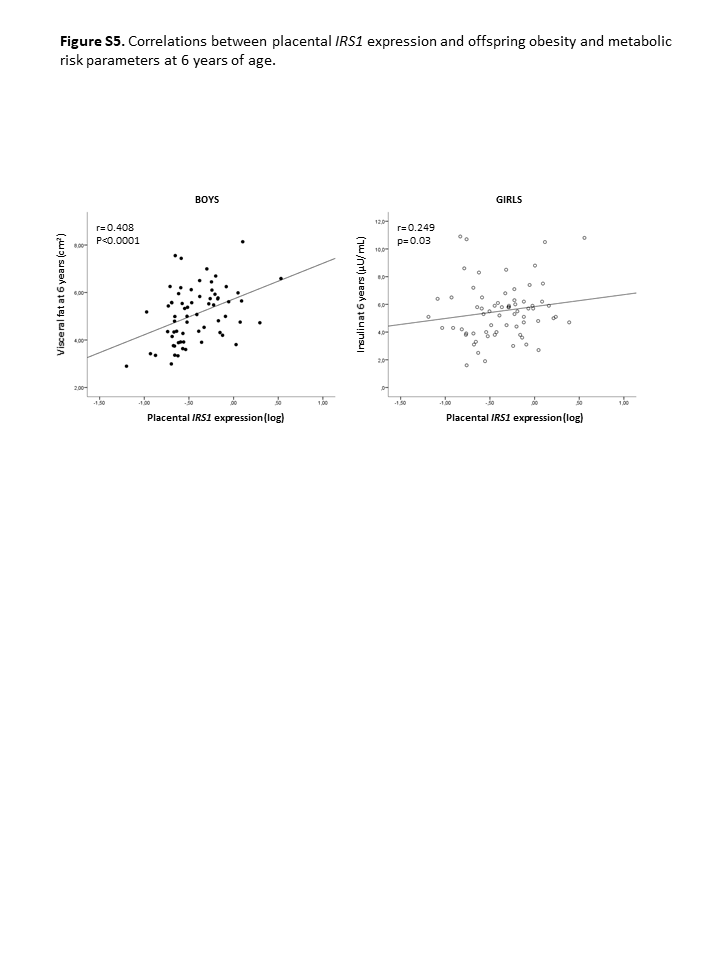

Supplement: Supplementary file 1 [file ijms-26-03141-s001.zip › Figure S5.tif]

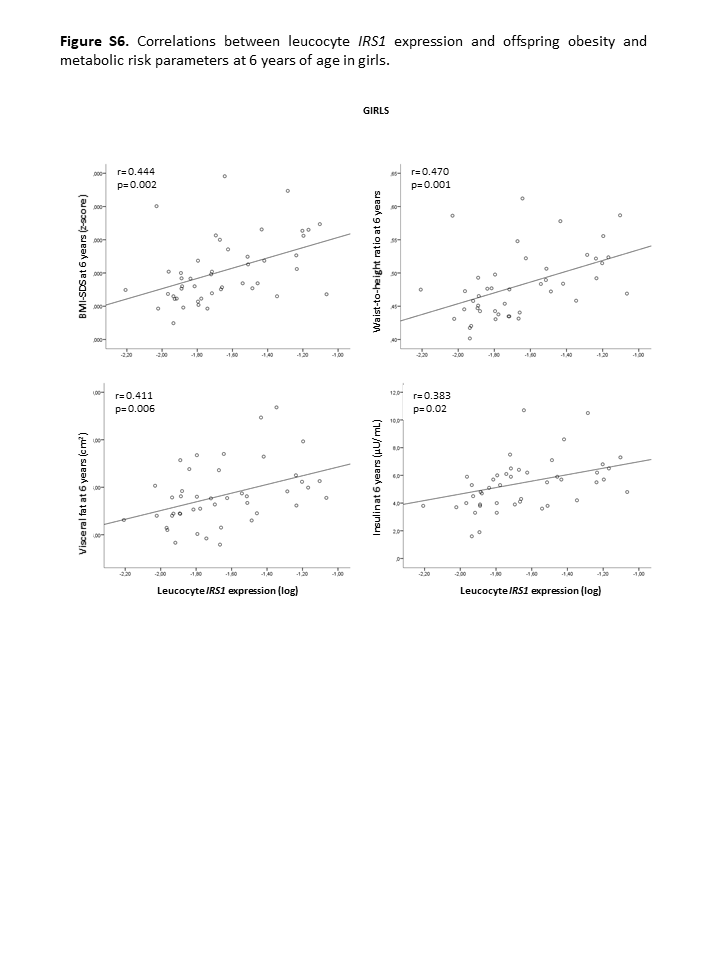

Supplement: Supplementary file 1 [file ijms-26-03141-s001.zip › Figure S6.tif]
